# COVID-19 and Its Sequelae Masquerading as Gastrointestinal Ailments: A Report of Gastroesophageal Reflux Disease (GERD) and Review of Recent Cases

**DOI:** 10.7759/cureus.57617

**Published:** 2024-04-04

**Authors:** Faris Shweikeh, Gordon Hong, Sana Rabeeah, Usma Shabir, Aijaz Sofi

**Affiliations:** 1 Internal Medicine, Cleveland Clinic Akron General, Akron, USA; 2 Internal Medicine, University Hospitals Cleveland Medical Center, Cleveland, USA; 3 Internal Medicine, The University of Toledo Medical Center, Toledo, USA; 4 Digestive Disease and Surgery Institute, Cleveland Clinic, Cleveland, USA

**Keywords:** long covid, pulmonary embolism (pe), esophageal disorder, gastrointestinal (gi), sars-cov-2, covid-19, #gastroesophageal reflux disease (gerd)

## Abstract

Coronavirus disease 2019 (COVID-19) predominantly causes respiratory symptoms. However, a rare segment of patients recovering from COVID-19 may develop gastrointestinal (GI) symptoms. We describe a case of a female who presented with symptoms suggestive of refractory gastroesophageal reflux disease (GERD) for 18 months following COVID-19 infection. Her symptoms included epigastric and chest pain, coughing, and vomiting. Upper endoscopy and 24-hour pH monitoring were negative. Following hospital admission due to worsening symptoms, she was diagnosed with chronic pulmonary embolism (PE) presumed to be related to COVID-19. Her reflux symptoms resolved within two days of the initiation of anticoagulation. Our findings suggest that chronic PE should be considered in patients presenting with GERD refractory to treatment following COVID-19 infection. Generally, as COVID-19 and its sequelae may masquerade as GI conditions, they should be on the differential diagnosis, especially in the post-pandemic era when routine testing has significantly declined.

## Introduction

The coronavirus disease 2019 (COVID-19) infection primarily results in respiratory symptoms and manifestations such as cough, shortness of breath, phlegm, congestion, and rhinorrhea [1]. However, gastrointestinal (GI) symptoms have been reported to occur in approximately 17% of patients [1]. The most common symptoms reported are anorexia, nausea, diarrhea, abdominal pain, and vomiting [2,3]. The pathogenesis could likely be due to known mechanisms such as an exaggerated inflammatory response and a highly coagulopathic state [4,5]. Of note, unknown mechanisms of pathogenesis of COVID-19 could also be an underlying cause and need to be further explored. Whatever the cause may be, the GI manifestations of the virus should be recognized. 

As our understanding of this disease has grown, we now realize that COVID-19 and its presentations can be much more complex than just a respiratory syndrome. Unusual presentations have been reported both during acute presentation as well as post-infection sequelae of the disease [6,7]. We present a case of chronic pulmonary embolism (PE) following COVID-19 infection, initially presenting as refractory gastroesophageal reflux disease (GERD). We hypothesize that two aspects of COVID-19 make the diagnosis and treatment particularly challenging: the associated pro-inflammatory process and hypercoagulable state. We also engage in a review of the literature on COVID-19 cases mimicking GI disease.

## Case presentation

A 64-year-old woman presented to her family practitioner for an evaluation of her new-onset GERD symptoms. The symptoms had begun after an episode of COVID-19 infection 18 months prior. She presented with complaints of epigastric and midsternal chest pain, associated with bouts of coughing, followed by vomiting. Her symptoms were more severe at night when lying down. She denied dyspnea. She was initially prescribed a proton pump inhibitor (PPI), omeprazole 20mg, once daily; however, this did not improve her symptoms even after up-titrating the dose. Physical examination was unremarkable, including lung and abdominal exams. Laboratory evaluation revealed a normal complete blood count and complete metabolic panel. She was referred to our GI clinic for esophagogastroduodenoscopy (EGD), which was normal. Subsequent referral to otolaryngologist did not reveal any head or neck abnormality to explain her symptoms.

**Figure 1:**
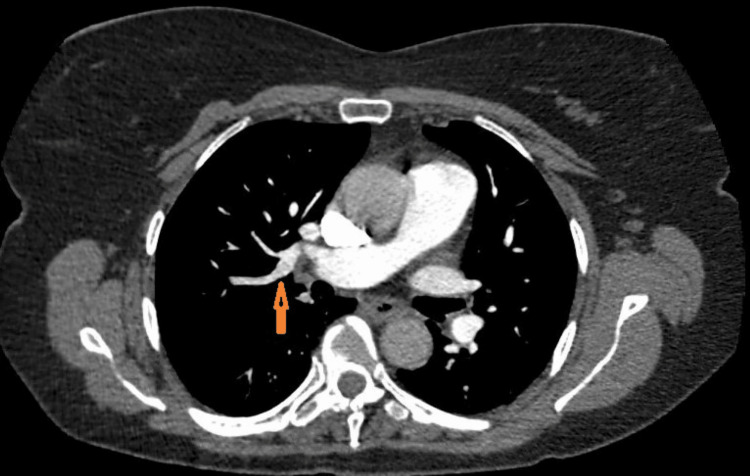
Pulmonary embolus seen on chest CT scan (arrow) CT: computed tomography

Given the poor response to PPI and negative endoscopic workup, we decided to perform a 24-hour Impedance pH study, which had a negative correlation of reflux events with her symptoms. She was admitted to the hospital four weeks later with an episode of severe bout of coughing associated with shortness of breath. She underwent chest CT, which showed multiple PE with involvement of the peripheral right and left pulmonary arteries and extension into multiple lobar branches (Figure 1). She was then started on a heparin infusion. Her cough and shortness of breath subsided two days later. She was then transitioned to apixaban and discharged home with the resolution of virtually all her GERD symptoms at follow-up. However, she was readmitted to the hospital for an evaluation of sudden-onset weakness of the left lower extremity 10 days after discharge and underwent a CT of the pelvis, which showed a hematoma on the left side of the coccyx. Apixaban was discontinued and she underwent emergent surgical drainage. She had a recurrence of chest pain with a cough followed by vomiting 24 hours after the discontinuation of apixaban. She underwent a subsequent placement of an IVC filter and apixaban was resumed six days later. Her GERD-like symptoms - including nocturnal and positional epigastric/chest pain, coughing, and vomiting - resolved two days after the resumption. She has remained asymptomatic for more than a year thereafter.

## Discussion

Several studies have indicated an association of coagulopathy in patients with COVID-19 infection [4,5,8-9]. The pooled incidence of venous thromboembolism (VTE) and PE in a systematic review of 35 observational studies from across the world (involving 9,249 hospitalized patients) was 21.6% and 11.8%, respectively [8]. Severe inflammatory response associated with COVID-19 infection by endothelial dysfunction, endothelitis, and elevated levels of prothrombotic factors has been postulated to lead to a hypercoagulable state [4,5]. In addition, pulmonary embolisms occurring late after recovery from acute COVID-19 infection have often been reported [9]. Unexplained chronic cough is also a rare manifestation of PE [10].

GI symptoms are known to occur in 29% of patients six months after recovery from COVID-19 infection, and, among these, reflux symptoms were reported in 27% [11]. In an Indonesian survey comprising 9,800 patients, it was found that there was an increased rate of GERD during the pandemic compared to the pre-pandemic period (67.9% vs. 61.8%, respectively, p<0.001) [12]. Other investigators attribute the worsening GERD symptoms to the restrictive lockdown measures during the pandemic [13]. New-onset GERD symptoms post-COVID-19 infection may last over a year and may persist for the long term [14]. Theories propose that SARS-CoV-2 infection-associated GERD is due to gut dysbiosis, cytokine storm, and effects of COVID-19 medications [15,16]. Moreover, autonomic dysfunction is a likely contributor, particularly in those with post-COVID-19 GI symptoms [17,18].

Our patient, on the other hand, initially presented with symptoms suggestive of GERD post-COVID-19 infection. She denied symptoms typical for PE, i.e., dyspnea, until late in the course of her disease. Delay in the diagnosis of PE is known to occur when the symptoms and signs are nonspecific [19]. Surprisingly, all her symptoms resolved within 48 hours of the initiation of anticoagulation therapy. Furthermore, the GERD symptoms recurred once the anticoagulation therapy was discontinued. Given these findings, chronic PE was deemed the most likely underlying etiology for her symptoms. Thus, our case is unique in that a known sequela of COVID-19 (i.e., PE) resulted in a long-term GI ailment (i.e., GERD) that resolved following appropriate treatment. 

Our review of the literature revealed cases in which COVID-19 can masquerade as various GI conditions (Table 1). It has been reported to mimic presentations of acute abdomen and appendicitis [20-22]. These cases, sometimes even those involving surgical intervention, are associated with the pro-inflammatory and hypercoagulable state [20,21]. Ashcroft et al. have described a patient with right-sided abdominal pain, reduced appetite, and fever [2]. Acute appendicitis was considered the culprit; however, CT imaging revealed sequelae of likely COVID-19 infection. More recently, a report discussed a case of post-COVID-19 multi-system inflammatory syndrome (MIS) with the chief complaint of abdominal pain, which was successfully treated following its recognition [23]. In another case of COVID-19 without suggestive respiratory symptoms, a middle-aged female with a history of remote Roux-en-Y gastric bypass presented with nonspecific abdominal pain, anorexia, and absence of flatus suggestive of internal hernia [3]. Imaging was unremarkable in the abdomen, but chest CT was indicative of lung ground-glass opacities suggestive of COVID-19.

**Table 1:** Summary of recent reports of COVID-19 cases masquerading as GI ailments or conditions MIS: multi-system inflammatory syndrome; IVIG: intravenous immunoglobulin; CBD: common bile duct; ERCP: endoscopic retrograde cholangiopancreatography; SBO: small bowl obstruction; NR: not reported; AIH: autoimmune hepatitis; LMWH: low-molecular weight heparin; PE: pulmonary embolus; RYGB: Roux-en-Y gastric bypass

Study	Initial diagnosis	Final diagnosis	Age, years	Sex	Procedures	Treatment	Outcomes
Baker and Krawitz, 2023 [23]	Acute surgical abdomen	COVID-19 MIS	32	Female	None	IVIG and IV methylprednisolone	Discharge on day 17
Khonsari et al., 2023 [24]	Cholangiocarcinoma	COVID-19 with CBD involvement	54	Female	ERCP	Sphincterotomy, CBD stent	Discharge and recovery
Agrawal and Harsh, 2022 [1]	SBO with perforation; mesenteric growth	Acute pancreatitis in the setting of COVID-19	72	Male	Exploratory Laparotomy	No further treatment following surgery	NR
Kulkarni et al., 2022 [25]	Acute on chronic liver failure	AIH flare in the setting of COVID-19	50	Male	None	IV methylprednisolone	Discharge on day 12
Malbul et al., 2021 [21]	Appendicitis	Acute appendicitis due to COVID-19	25	Male	Appendectomy	COVID-19 management	Discharge on day 14
Ugolotti et al., 2021 [26]	Intrahepatic cholangiocarcinoma	Vascular injury due to COVID-19	68	Male	Liver biopsy	LMWH, aspirin for thrombi, PE	Recovery after 1 month
Suwanwongse and Shabarek, 2020 [22]	Appendicitis	COVID-19	18	Female	None	Supportive care	Discharge and recovery
Ashcroft et al., 2020 [2]	Appendicitis	COVID-19	NR	Male	None	Supportive care	Discharge and recovery
Betton et al., 2020 [3]	Internal hernia after RYGB	COVID-19	57	Female	None	Supportive care	Discharge on day 2

In 2022, Agarwal et al. reported the case of a COVID-19 patient who presented with acute pancreatitis, under the hypothesis that pancreatic injury is the result of SARS-CoV-2 virus entry via angiotensin-converting enzyme 2 (ACE2) receptor [1]. Early in the pandemic, a patient with a history of autoimmune hepatitis (AIH) and recent travel presented primarily with symptoms of liver failure including jaundice, ascites, and transaminitis [25]. The patient’s SARS-CoV-2 test was also found to be positive and the authors suggested the possibility of AIH flare in this setting. Two other cases have been described in the setting of COVID-19 presenting with symptoms and workup resembling cholangiocarcinoma attributed to viral-induced cholangiopathy and portal vessel damage [24,26]. Finally, recent articles evaluating long COVID-associated GI symptoms/conditions have proposed alternate mechanisms for their occurrence, such as residual replicative virus particles in GI and hepatic tissues, changes in the gut microbiome, and autoimmune processes, resulting in autonomic dysfunction including POTS-like presentations and/or GERD [19,20,27].

## Conclusions

COVID-19 infection and its manifestations can mimic various GI conditions, and a diagnosis can be made by the recognition of this possibility with a high index of suspicion. While GERD may occur following COVID-19 infection in some patients, clinicians should consider chronic PE in those with recurrent and recalcitrant symptoms of GERD post-COVID-19. This is even more pertinent when multiple standard evaluations and testing for GERD are inconclusive. Thus, in such situations, a workup for PE (e.g., D-dimer, CT imaging of the chest, etc.) should be considered. Furthermore, in the post-pandemic world, we have seen a decline in routine testing for COVID-19. However, testing acquires great importance when considering the various GI presentations of COVID-19. Clinicians should include this condition in their differential diagnosis and also query about previous infections in a particular patient, as treatment of it and its sequelae most often results in good outcomes and prevents an uncertain management course and unnecessary procedures. Future research should focus on the pathogenesis of GI manifestations of this mysterious virus.
